# How does the WHO Surgical Safety Checklist fit with existing perioperative risk management strategies? An ethnographic study across surgical specialties

**DOI:** 10.1186/s12913-020-4965-5

**Published:** 2020-02-12

**Authors:** Hilde Valen Wæhle, Arvid Steinar Haugen, Siri Wiig, Eirik Søfteland, Nick Sevdalis, Stig Harthug

**Affiliations:** 1 Department of Research and Development, Haukeland University Hospital, Jonas Liesvei 65, N-5021 Bergen, Norway; 2 Department of Clinical Science, Faculty of Medicine, University of Bergen, Bergen, Norway; 3 Department of Anaesthesia and Intensive Care, Haukeland University Hospital, Bergen, Norway; 4 Centre for Resilience in Healthcare (SHARE), Faculty of Health Sciences, University of Stavanger, Stavanger, Norway; 5 Department of Clinical Medicine, Faculty of Medicine, University of Bergen, Bergen, Norway; 6 Centre for Implementation Science, Health Service & Population Research Department, King’s College London, London, UK

**Keywords:** Surgical safety checklist, Patient safety, Ethnography, Quality improvement, Health services research

## Abstract

**Background:**

The World Health Organization (WHO) Surgical Safety Checklist (SSC) has demonstrated beneficial impacts on a range of patient- and team outcomes, though variation in SSC implementation and staffʼs perception of it remain challenging. Precisely how frontline personnel integrate the SSC with pre-existing perioperative clinical risk management remains underexplored – yet likely an impactful factor on how SSC is being used and its potential to improve clinical safety. This study aimed to explore how members of the multidisciplinary perioperative team integrate the SSC within their risk management strategies.

**Methods:**

An ethnographic case study including observations (40 h) in operating theatres and in-depth interviews of 17 perioperative team members was carried out at two hospitals in 2016. Data were analysed using content analysis.

**Results:**

We identified three themes reflecting the integration of the SSC in daily surgical practice: 1) Perceived usefullness; implying an intuitive advantage assessment of the SSCʼs practical utility in relation to relevant work; 2) Modification of implementation; reflecting performance variability of SSC on confirmation of items due to precence of team members; barriers of performance; and definition of SSC as performance indicator, and 3) Communication outside of the checklist; including formal- and informal micro-team formations where detailed, specific risk communication unfolded.

**Conclusion:**

When the SSC is not integrated within existing risk management strategies, but perceived as an “add on”, its fidelity is compromised, hence limiting its potential clinical effectiveness. Implementation strategies for the SSC should thus integrate it as a risk-management tool and include it as part of risk-management education and training. This can improve team learning around risk comunication, foster mutual understanding of safety perspectives and enhance SSC implementation.

## Background

The World Health Organizationʼs (WHO) Safe Surgical Checklist (SSC) [1] has been advocated globally, and in some cases mandated as a surgical safety intervention, aiming to improve information exchange within the perioperative team, and to critically review specific safety items [2]. Clinical effectiveness studies have demonstrated beneficial impact of the SSC implementation on a range of patient- and team outcomes, including mortality rates, complication rates, length of in-hospital stay, teamwork, and adherence to safety processes [3–10]. Also, high-fidelity use of the SSC, i.e. suitable use of all three parts of it, has been shown of crucial importance in order to achieve improved outcomes [11]. The evidence thus supports that high quality implementation of SSC is required for positive effects to be attained [12].

Studies on the implementation of the SSC, however, have had mixed results [13, 14]. Further, research shows that the SSC is sometimes used patchily, and that SSC implementation quality differs among hospitals, surgical specialties, surgical staff members, and among specific items and parts of the checklists [15–18]. In addition, longitudinal implementation studies of the SSC have offered only modest, sustained impacts on staff attitude- and satisfaction, and surgical team perspectives [19–22]. Instead, conflicting findings and failings to link the SSC to improved outcomes are causing some at least scepticism around its true potential as a patient safety intervention [15]. Questions on how lack of SSC compliance might actually introduce new risks not present before have also been raised [23], prompting calls for the reconsideration of policies mandating the SSC as an organisational safety practice [24].

Although variations in SSC fidelity of use have been documented, there is limited understanding of why such variations occur [25–28]. Safety interventions, their implementation and the clinical and organisational context within which they are applied are intertwined and mutually interacting, thus influencing how such interventions actually work in practice (or not) [29]. Structural changes in operating staff workflow and their perceptions of the SSC and patient safety are reccomended to improve SSC implementation [25].

Ultimately, the reduction of risk SSC aims to achieve is not achieved by ‘ticking off’ checklist items, but by the actions and behaviours of the perioperative team the SSC calls for. [27] A knowledge gap still remains of how perioperative staff integrate (or not) the SSC into their pre-existing risk management strategies and tools; and how their risk perceptions are impacted by the use of the SSC. Studies that seek to understand the role of adaptive, human and social practices in safety efforts such as the SSC are therefore called for [30–32].

Reflecting on the purpose of the SSC, we propose that for a safety intervention aiming at human behaviour, it is essential that all team members share an understanding of clinical risk and risk management strategies; and that the intervention is actually embedded effectively and efficiently into existing safety practices. Thus, the aim of this study was to explore how the multidisciplinary perioperative team members integrate the SSC as part of their risk management strategies in perioperative care.

## Methods

### Design

This is a prospective ethnographic study. Multidisciplinary perioperative teams were observed during performance of the SSC in operating theatres (OTs), followed by face-to-face interviews of key informants. While focusing on description and analysis of “everyday” routine practice in their natural settings, this design is well suited to capture both participants’ use of SSC and risk communication patterns, as well as their perceptions of patient safety challenges [32, 33].

### Study setting

**Fig. 1:**
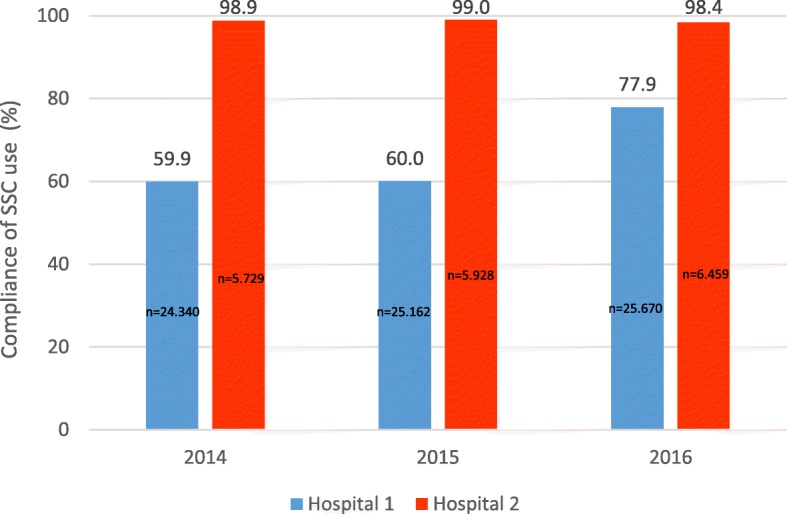
Longitudinal monitoring of SSC compliance rates in surgical procedures (n = total numbers of procedures/hospital/year) performed between 2014 and 2016 for study hospital 1. (tertiary teaching hospital) and study hospital 2. (central community hospital)

The study was conducted in two hospitals, a tertiary teaching hospital and a central community hospital, within one of the four Regional Health Authorities in the country. Hospital characteristics are described in Table 1. The hospitals operate within separate organisational structures, and perioperative routines vary accordingly. One surgical unit at each hospital was included in the study. These hospital units served as surgical study-clusters in a large stepped wedge, cluster randomised control trial of the WHO SSC’s impact on patient outcome in 2009–2010, and were therefore recruited [8]. The adapted national version of the WHO SSC had been implemented at both the surgical units, following an educational program with standardised lectures and dissemination events [22]. Generally there were customisations of the SCC as recommended by the WHO at the two hospitals, with additional department level customisations in the tertiary teaching hospital. These customisations were individual and not coordinated or consistent, but according to local routines and practice. Following initial introduction, SSC utilisation was monitored by both the local hospitals and the Regional Health Authority, as part of the national Patient Safety Programme: In Safe Hands, commissioned by the Ministry of Health and Care Services [34]. The observed SSC utilisation indicator was defined as: number of surgeries where the SSC was used over total number of performed surgeries [34]. Longitudinal monitoring of SSC compliance data from 2014 to 2016, showed differences between the two hospitals (Fig. 1), such that compliance was lower for hospital 1 compared to hospital 2.

**Table 1:** Hospital and interviewee characteristics

HOSPITAL CHARACTERISTICS					INTERVIEWEES CHARACTERISTICS					
	Size^**a**^	Surgical hospital stays^**b**^	Level	Organisational structure	Number *N* = 17	Nurses^**c**^ Nurse anaesthetist/ Operating theatre nurse	Physicians^**d**^ Consultant anaesthesiologist/ Consultant surgeon/Surgeon	Cardiovascular perfusionist^**e**^	Sex female/ male	Work – experience years qualified in profession - range
Hospital 1:	1066	33,584	Tertiary referral hospital	22 specialised units	9	4	3	2	4/5	5–32
Hospital 2:	244	7887	Secondary referral hospital	2 specialised units	8	4	4	0	3/5	3–30
Total	1310	41,471	–	–	17	8	7	2	7/10	3–32

^a^Size: 2016 Occupancy rate (Statistics Norway) = bed-days/available bed-days. ^b^Surgical hospital stays: 2016 reported stays with one or more surgical procedure, based on the classification system of the Norwegian diagnosis related groups (N-DRG, Norwegian Patient Registry. ^c^Authorisation requirements in Norway: 3-year bachelor degree in Nursing-180 ECTS^§^ + either a 1,5-year Specialist education program-90 ETCS, or a 2-year Master’s program-120 ECTS at a College University degree. ^d^Authorisation requirements in Norway: 6-year cand. Med degree, 360 ECTS + 6,5 years of specialist training before qualification as consultant. ^e^Authorisation requirements in Norway: 3-year bachelor degree in Engineering or Nursing180 ECTS + a 2-year Master’s program-120 ECTS at a College University degree. ^§^European Credit Transfer and Accumulation System (ECTS) credits

### Data collection

Data collection involved non-participant observations and interviews together with longitudinal SSC compliance rate reports derived from administrative data systems (described in detail below). Data triangulation was used across time, hospital settings and professional groups, to capture a contextualised ‘portrait’ of the SSC within the studied settings [35, 36].

#### Perioperative observations

We observed 6 complete surgical cases at each of the study sites. Observations took about 1 week per site, and covered specialties of general- and highly specialised surgery. The observations (40 h) covered scheduled surgical procedures at dates agreed upon beforehand by the service managers. All cases were elective, done under general anaesthesia during normal working hours, and covered both complex cases and day-surgeries. Cases where any staff member or the patient withheld consent were excluded. The observations aimed to map routine behaviours of “work as done”, ie. actual SSC team performance, which differs from the concept “work-as-imagined” (how it should have been done), as the latter cannot capture context and nuances of clinical work or how circumstances vary [37].

The checklist was initially introduced to the hospitals in a randomised controlled trial in 2009–2010, as described by Haugen et al. [8] The data for this study were collected in 2016 at one hospital at a time, with team observations taking place prior to interviews, starting at the central community hospital (hospital 2 in Table 1). The data collection at the tertiary teaching hospital was carried out a couple of months later, as the process of recruiting informants took slightly longer time. Observations of team interactions and communications were noted and reviewed by the research team. These field notes were used to develop the interview topic guide and inputs to the data analysis.

#### Interviews

Interviews were carried out with 17 members of the perioperative teams, each typically consisting of one or two surgeons, at least two operating theatre nurses, one anaesthesiologist, one or two nurse anaesthetists, and occaisionally one cardiovascular perfusionist. Interview topics covered SSC use, team-work and communication patterns (interview guide in Additional file 1). All healthcare personnel in the perioperative team were considered key informants. Hence, a maximum variation purposive sampling strategy [38] was used to elicit professional perspectives on SSC utilisation in the OTs. Invitations to participate were initially reviewed and approved by hospitals managers at the respective study hospitals. Participants were recruited by the surgical unit managers. Professionals with variable length of perioperative work experience were targeted for sampling; their characteristics are described in Table 1. All interviews were conducted in the OT departments, in areas free from distractions (e.g., meeting rooms). Each participant was interviewed once. The interviews lasted between 28 and 47 min, with median length 36 min. The interviews were audiotaped, and transcribed verbatim for analysis.

All observations and interviews were performed by HVW (MSc, senior nurse anaesthetist, trained in qualitative research). A second researcher, ASH (PhD, senior nurse anaesthetist, trained in qualitative research) participated in 6 h of the observations to ensure trustworthiness of the findings.

### Analysis

Data from observations and interviews were analysed using an inductive, content analysis approach [39]. The following steps were used: HVW, ASH, SW (senior safety scientist, trained in qualitative methods), and SH (quality manager and senior scientist), read the transcribed interviews forming units of analysis. HVW identified and coded transcript sections into ‘meaning units’, followed by relating categories and themes, constituting the manifest content [39]. Observational data were used to support the interview data analysis and contributed to the formation and interpretation of the latent content, and emerging themes. ASH, SW and SH reviewed the coding and interpretations. Preliminary themes, subthemes and quotes were then discussed amongst all authors, using group consensus to strengthen coherence of the findings [40]. The finalised dataset is presented in emerging themes.

## Results

Analysis of observations and interviews identified three major themes: (1) Perceived usefullness, (2) Modification of implementation, and (3) Communication outside of the checklist. In the following sections, each of the themes are presented in detail. The identified themes and corresponding categories are presented in Table 2, with representative verbatim quotes and observation notes (in italics) to illustrate the findings.

**Table 2:** Themes and categories with illustrative participant quotes and observation notes (in italics)

THEME	CATEGORY	ILLUSTRATIVE QUOTES FROM PARTICIPANTS (Observation notes in italics)
**Perceived usefullness**	Lack of practical utility	**Anaesthesiologist:** Before I anaesthetise the patient, I know all the parameters for my patients, I check their circulation, and I know about their vascular occlusions and specific arterial stenosis, and I feel I have complete control of the patient, so …. It is hard to think that the checklist will provide extra safety for me. **Anaesthesiologist:** Patient safety is part of our training as anaesthesiologists from the very beginning! Eh- check of the anaesthesia machine, instruments, the patients, and practically checks of everything we do! Double control of every blood products provided, medications, everything! In addition to assessing the patient in person and talking to them prior to surgery. We have always performed these items; it is part of the standardised pre-operative anaesthesia assessment and preparations. **Nurse anaesthetist:** The anaesthesia machine is not due to any variation, it should be checked prior to every anaesthesia. We do not admit patients into the OT unless the anaesthesia machine is OK. **Surgeon:** Well, the SSC has a function, in a very simplistic way, but it does not have a proper control function, the way it is supposed to, because we have so many checks and control mechanisms incorporated. So, I don’t think that the SSC is as important to us, as to other surgical departments, which have other pre-operative assessment routines. We have so many points of assessment, where our patients are discussed and evaluated.
	Perceived benefits	**Operating theatre nurse:** The SSC is useful as a reminder of double checks of labelling tissue samples, and to make sure the right surgical equipment is present. Surgical routines are complicated when you are a beginner … **Nurse anaesthetist:** I value how the SSC may contribute in aligning the surgical and anaesthesia plan for the entire team. **Surgeon:** The team introduction is a nice way to start team working; the “Time-Out” is in a way a mental team-calibration.
**Modification of implementation**	Review and confirmation of items	**Cardiovascular perfusionist:** And occasionally, I may have to call out if there is something I believe is required or something has been omitted, i.e. that the patient has low haemoglobin levels, and I need to take action. In addition, during haemodilation, I avoid infusing too much fluid in the machine. Then I tell the surgeon and anaesthesiologist what I intend to do, to make them understand what I intend to do. **Operating theatre nurse:** Some surgeons that are more reluctant than others, they just start to mumble through the SSC as soon as they enter the OT, and then proclaim to have performed time-out. Then, it is required from an OT nurse to be determined and speak up, and say, «no, this is not good enough! Everybody needs to know what you just said! » Sometimes I have to add: «No, this was not loud enough, you have to repeat the SSC! » However, to speak up requires some years of work experience. **Operating theatre nurse**: I think the SSC is a good thing, but I miss team concentration during its performance Things have improved, from the beginning until now, but there is still too much disturbance during SSC performance. I really miss that everybody stops and pays attention. Due to the workflow in the OT, there is always someone who pursuits some kind of work, and does not stop. In addition, you need to pay full attention for the SSC to be advantageous. **Nurse anaesthetist:** But it is obvious, the SSC performance is totally depending on the physicians participation. As soon as they became more involved, both performance and compliance increased.
	Presence of team members	**Nurse anaesthetist:** Personally, I prefer to perform the sign-in with the anaesthesiologist being present in the OT, I think it is embarrassing to repeat the questions and items I have asked the patient previously, upon arrival in the OT. So I have almost stopped to ask the patients about their potential allergies, and so on. The anaesthetist repeats everything when they arrive in OT anyway. ***Observation:*** *The team compositions varied during the different parts of the SSC performance; The nurse anaesthetist, operating theatre nurse and anaesthesiologist were present during “Sign-In”. The nurse anaesthetist, operating theatre nurse, surgeon(s) and anaesthesiologist (occasionally) were present during “Time-Out”. The nurse anaesthetist, operating theatre nurse, surgeon(s) and anaesthesiologist (occasionally) were present during “Sign-Out”.*
	Barriers of performance	**Nurse anaesthetist:** Well, you don’t want a conflict within the OT, you’re in a way a bit tired of that, so you try once more to perform the SSC, and if you do not receive any attention, you just let it go and tick off the box, even though it has not been performed. **Nurse anaesthetist:** It is so important to keep the SSC short, because it does in a way disturb our workflow.. You are about to start induction of anaesthesia, and then; «No, no, we have to stop and perform the SSC! » Our workflow is interrupted, and it is very disturbing and frustrating. **Operating theatre nurse:** The anaesthesia team is responsible for the anaesthesia, medications …. It is their responsibility**.** Questioning them about this is like questioning them whether they have done their job or not. … .
	Registration practices	***Observation:*** *At the surgical units in hospital 2, SSC performance was ticked off either after “Sign-In”, or the “Time-Out” part. There was only one box that needed to be ticked off electronically, in order for the SSC to be registered as performed. At the surgical unit at hospital 1, all three parts of the checklist had to be ticked off as three separate boxes in order for the SSC to be registered as performed.*
**Communication outside of the checklist**	Patient specific risk communication	**Anaesthesiologist:** In general, we have contact with the cardiovascular perfusionist prior to surgery, to inform them about patient specific details such as medications, because they don’t read the patient records the same way we do. **Operating theatre nurse:** …. And if bleeding is involved, we need to notify the anaesthesia team about the estimations of blood volume collected in the surgical suction, before other fluids are added.
	Selected communication of risks	**Cardiovascular perfusionist**: … and these preparations are being discussed between the surgeon and the cardiovascular perfusionist prior to surgery. **Operating theatre nurse:** In most cases, we have direct communication with the anaesthesiologist during induction of anaesthesia, and ask permission to start our preparations, such as positioning the patient, or inserting the urinary tract catheter. **Anaesthesiologist**: … and then, the surgeons talk about the details of the surgery they have performed, while rushing out of the OT, right? And then you have to talk with them afterwards anyway, due to potential considerations post-operatively, like the follow-up antibiotic prophylaxis. Then you have to initiate contact anyway, because certain things require a follow-up.

### Perceived usefullness

Participants expressed various views related to SSC’s practical utility. The anaesthesia team (nurse anaesthetists and anaesthesiologists) perceived the SSC to lack practical value, especially the “Sign-In” part, which was perceived as not adding anything new to reduce anaesthetic risk. They reported that they had good control of procedures and tasks before induction of anaesthesia. Existing checking mechanisms and protocols were considered sufficient, as pre-anaesthetic patient risk assessments; e.g. difficult airways, medications, allergies were performed in advance, and safety tests and -checks of the anaesthesia machine, − equipment and -medications, were incorporated in existing routines and reviewed prior to induction of anaesthesia. Checks performed by the anaesthesia team during the preoperative phase were aligned with their roles and responsibilities, acknowledged by both the anaesthesia team and other perioperative members. In addition, some anaesthesiologists expressed a need of retrieving surgical information regardless of the SSC, which in their opinion made reviews of SSC “Sign- In” items superfluous. Yet, some anaesthesiologists expressed a need for more time to review and handle high-risk patients together with the nurse anaesthetists, during a pre-anaesthesia briefing.

Interestingly, however, other staff-members described situations where they experienced the SSC as being particularly useful i.e.; by confirmation of patient identity, as a reminder-list of important safety checks, especially for procedures that might vary according to types of surgeries, or patient specific conditions such as administration of surgical antibiotic prophylaxis. OT nurses described how surgical equipment reviews during “Time-Out” were advantageous, as well as tissue-sample labelling double checks at “Sign-Out”. SSC was also highly valued in order to provide predictability in the OT, e.g., logistics in OT scheduling, timing of anaesthesia, and for preparation and reports to post-anaesthesia ward. Nurses in particular, reported an ease of workflow when everybody in the team knew the surgical plan. In addition, the “Sign-Out” provided a sum-up of the surgery, which were reported being of help to understand exactly what procedures that had been performed. This was considered helpful in correct surgical procedure codings. Introduction of the team members during SSC “Time-Out” was also described by some surgeons as unifying the team to structure their focus before incision. This was especially useful for new and/or unexperienced team-members.

### Modification of implementation

Observations identified variations in how different items and parts of the SSC were carried out – and also in how the electronic registration of the SSC was done (the latter is important as it is used to provide national compliance rates). Policy for hospital 1 mandated specific registration of each of the three parts of the SSC (so 3 separate registrations) whereas policy for hospital 2 mandated one SSC registration including all three parts (so 1 registration in total).

SSC utilisation varied across different SSC items and participants’ perception of challenges of actual use. Observations showed that induction of anaesthesia done in the OT in both units silenced and concentrated the team members present in OT. Yet, performance of the SSC “Sign-In” only few minutes earlier did not have at all the same effect: it failed to concentrate the teams’ attention.

Participants described how verbal SSC briefings rushed through the items, forgetting to include the whole team. Lack of team focus- and concentration during SSC performance was also described. When SSC checks interfered with existing workflow, the SSC was often partly or poorly performed, delayed, or left out as a result. Resistance within the team and verbal disturbances also influenced performance. As a result, SSC registration was often described as a “tick-off exercise”, which some of the participants vocally worried about its impact on safety.

Presence of the different team members in the OT also influenced how- and by whom the SSC items were checked. While nurse anaesthetists and OT nurses were present during all three parts of SSC, surgeons and cardiovascular perfusionists were not present in OT during “Sign-In”. Cardiovascular perfusionists also described being haphazardly included or not during “Time-Out”, unless they actively initiated communication themselves about specific items or equipment in use. Anaesthesiologists described that their presence in OT during “Time-Out” and “Sign-In” was more relevant in complex surgical cases, and for high-risk patients.

### Communication outside of the checklist

Risk communication and critical information exchanges during perioperative care were performed in multiple, formal and informal micro-team constellations. The team members’ individual and professional perception of identified or potential patient safety challenges influenced SSC utilisation, and how, when, and to whom information on risk was passed in the perioperative phase of surgery. Their perceptions of safety challenges also influenced how the team members viewed and exerted influence on risk communication within the team.

In one of the study sites, according to participants, formal team constellations featured preoperative morning meetings where the surgical schedule of the day was presented by the surgeons in charge. Relevant safety issues were discussed amongst the present team members. Team members who had been present at the meeting then disseminated information of importance to their respective colleagues. Some of the interview participants described this information exchange as a “sub-optimal, second hand ad-hoc information transfer”. Instead, they would have preferred that team briefings were better structured prior to surgery, involving the actual team members scheduled for that specific surgical procedure. Aligning the SSC items and reviews according to specific risks related to the individual patients and their specialties was also suggested.

The local SSC version was scaled down to cover a minimum of items. This was explained by physicians in charge as being sufficient, partly due to factors such as strong organisational structures, a limited variety of surgical procedures and standardised operative environment with few OTs. Moreover, the required competencies, professional experience and good inter-staff relationships were also cited as elements justifying the reduction of SSC content. This was emphasised in terms of the highly qualified and experienced multidisciplinary perioperative team members and local practice of one-to-one relationship between the anaesthesiologist and the patient, throughout the perioperative pathway.

The formal planning of surgery and anaesthesia was performed by the respective surgeons and anaesthesiologists in charge. If somehow concerns about the patient needed to be discussed more thoroughly, i.e.; clarifications about the procedure, required equipment, laboratory tests, blood products, or patient medications, the different health care personnel directly contacted the responsible professionals. This form of patient specific communication and information exchange within micro-team constellations was observed throughout the perioperative phase – such that:

- the anaesthesia team reported to have an on-going dialogue about the patients’ risks, necessary equipment, fluids and medications.
- the OT nurses and surgeons had a continuing dialogue on maintaining a sterile field, possible risks and lack of equipment, specimen labelling and compress counts.
- cardiovascular perfusionists, anaesthesiologists and nurse anaesthetists had an ongoing dialogue on collaboration of the haemodynamic controlling.
- the anaesthesiologist had also ongoing dialogue with the surgeon in charge.

These interactive patterns of micro-team communication and information exchange clearly dominated and superseded any SSC checks.

## Discussion

This study explored in detail how the perioperative team integrates use of the SSC as part of their risk management strategies in real time during patient care. The individual and professional “cost-benefit” assessments of practical usefulness of the SSC influenced which checks were given attention and by whom. Existing patterns of micro-team risk communication clearly took precedence over formal SSC utilisation.

Our findings correspond to the results of a global survey among medical professionals regarding the SSC [41]. Among the 6269 respondents, impression of usefulness (67%) was the main factor associated with the SSC usage [41]. The perceived (un) importance of checklist items influencing SSC use, was also found in a Canadian study [42]. How team members perceive SSC sense making in practice has further been related to the relevance of specific SSC items, and possibilities of tailoring SSC content to local context [25, 27, 43, 44].

Anaesthesiologists have previously been identified as being the least positively disposed towards SSC completions, when compared with surgeons and nurses [45]. We found that nurse anaesthetists and anaesthesiologists in particular reported that their existing safety protocols and procedures such as the pre-anaesthetic patient risk assessment were sufficient. The “Sign-In” review was seen as redundant, coinciding with former arguments of SSC performance being double checking routines [17, 42]. Still, this perspective raises the concern of overlooking other team-members’ possible information needs. It might also indicate that “perception of risk” is primarily concerned with a narrow view of active failures associated with one’s own professional role, rather that wider underlying conditions that impact upon the entire perioperative team [17]. Whilst the SSC is designed to reduce risk perioperatively, for it to work as a team-based intervention a shared understanding among all team members of this simple aim is important. In a previous study, we have reported that improved patient outcomes have been associated with improved care processes due to high quality use of the SSC [11]. This indicates the importance of ensuring that i.e., risk of hypothermia- and responsibilities of corresponding, preventative actions such as antibiotic prophylaxis is communicated with the team as a whole. If team members’ perceptions of risk are solely concerned with their professional perceptions of active failures instead of including underlying conditions, such as risk of developing surgical site infections, important safety aspects of the team communication are neglected [17, 46].

**Fig. 2:**
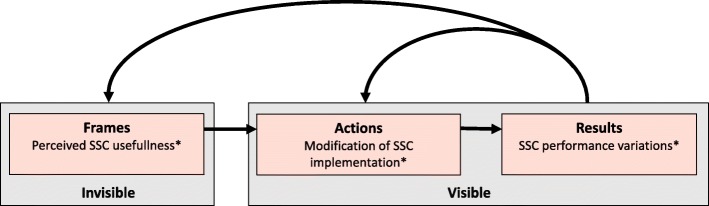
Revised model of “Reflected practice” based on: Rudolph J, et al. Simul Healthcare 2006;1: 49–55. “Frames”, “Actions” and “Results” are illustrated with examples* derived from results of the content analysis

In addition to the narrower and wider risk perceptions, we found that SSC utilisation is also a function of how it is incorporated into team membersʼ workflow schedules in OT, and how much effort has been spent reducing practical barriers within the team [47]. This finding corroborates previous investigations [18, 25, 42, 44]. However, we identified that the two study hospitals had different policies for how the SSC performance was registered and measured. This may explain some of the observed variation between the two hospitals. Also, variation in style of checklist implementation between the hospitals, the presence of local champions, differences in safety culture, the support and involvement of management, might account for the variation [18, 48]. In terms of these impactful factors, we suggest that SSC performance variations might offer distinct opportunities to address risk management at the intersection of perioperative procedures and actual team working. “Reflective practise” is a well-known method used to scrutinise oneʼs own taken-for-grated assumptions and professional work practice, often accomplished in a collaborative setting [49]. The theory underlying reflective practice draws on cognitive science and social psychology, and the central idea is that people make sense of external stimuli through internal cognitive “frames”. These invisible frames, in turn, shape the actions people take. Actions including speech, are observable as are most results. Figure 2, based on the “Reflective practice” model by Rudolph and colleagues [49] illustrates how the “invisible” perceived utility of the SSC influences actions of how the SSC implementation is modified, and further results in visible performance variations in an ongoing process. If hospital managers fail to regard the SSC as a complex, social intervention and instead exert demands for high compliance rates of SSC performance as a top-down approach, this can lead to workarounds and outright resistance, and cause for the checklist to be used as a tick-box exercise to meet management requirements [25, 50].

### Strengths and limitations

The use of an ethnographic design is well suited to capture “everyday” routine behaviours in their natural settings [32, 33]. By combining observations and interviews, participants were given opportunity to identify and share insights into observed practices of SSC performances that deviated from the norm. However, this study was limited to explore team perception of risk management strategies in relation to the three parts of the SSC, rather than each specific SSC item. How team members consider use of the SSC to match their perception of patient safety challenges in perioperative care, might therefore be limited to reflect local roles and responsibilities of teamwork practice. In order to achieve credible information, data triangulation was used by collecting data across time, hospital settings and professions [35]. Although all members of the multidisciplinary surgical team were represented by maximum variation purposive sampling strategy, interview selection bias remains a possibility. As both observers had anaesthetic background, this may have introduced biases into the observations.

Although we could not control for any unconscious such biases during the observation phase, the observation guide was reviewed and agreed upon with members of the research team, who had different professional backgrounds. Following completion of the observations and interviews, their coding and analysis was further reviewed and debated within the multiprofessional research team, to ensure balance of professional opinion.

### Practical implications and future directions

When well applied, the SSC is an effective intervention. It has been associated with relative risk reduction of 0.42 (95% confidence interval (CI), 0.33–0.50) of surgical complications, and significant reduction in length of in-hospital stay in a randomised trial [8]. A recent population cohort study from Scotland documented a reduction of 36.6% (95% CI 55.2–17.9) in mortality [51]. Whilst the clinical effectiveness has been shown, study of implementation strategies to address influential barriers to SSC usage is needed, coupled with studies of the implementation process and local contexts [25]. Our findings indicate that how the perioperative team members perceived SSC as a risk reducing intervention, has considerable impact on the execution of the SSC and risk communication around it. We therefore propose that the SSC needs to be explicitly integrated into the risk management toolkit of perioperative care. An incident analysis from one of the study hospitals recently reported that a patient had wrong surgery despite use of the SSC. One of the causes contributing to the adverse event was lack of team response to detected departures from planned care when the SSC was done [52]. This incident demonstrates that we need to move beyond use of SSC as a symbolic safety check; like other safety interventions, the SSC is vulnerable to meaningless application [23]. When the SSC is seen as an “add-on”, or more commonly conseptualised as an external “thing” [31], the challenge of its integration into perioperative work remains.

How does the SSC become better integrated as a perioperative safety strategy? We propose that the SSC needs to be formally established as one (and only one) element of our toolkit of standardised perioperative safety mechanisms. This will contribute to the development of a shared mental model within perioperative teams [53], such that the SSC becomes owned by them and applied in conjunction (and not in addition to) all other safety mechanisms in the OT, and indeed also pre- and post-operatively. This proposal follows on from recent policy developments in perioperative safety. For example, the national standard for the safe practice of anaesthesia, and the Helsinki declaration on patient safety in anaesthesiology [54] have established normative guidelines for everyone who provides anaesthesia care [55]. The observed behaviours related to induction of anaesthesia, reflect a sense of situation awareness amongst the team members, which might stem from a common understanding of this safety standard. In the UK, the National Safety Standards for Invasive procedures have been developed to set out the key steps necessary to deliver safe and common care standard for surgery, including the SSC but also many other checks and tools [56]. We believe that such a normative standardisation would contribute to establishing a shared mental model for the SSC globally. Of course further implementation strategies are required to translate standards into practice – including educational interventions, regular dissemination and updating of the standards based on emerging evidence [57].

## Conclusion

This study showed that when the SSC is perceived as an “add on” and not integrated as a risk management tool or part of the multidisciplinary risk management strategy, its fidelity is low. Strategies to enhance patient safety in surgery should focus on a multidisciplinary approach to foster shared mental models of safety standards in the OT. Aligning risk-assessment in SSC staff education where the SSC is part of a safe surgical risk assessment system, might provide an improved sense of value to all OT personnel, improve team learning of risk communication, and foster mutual understanding of safety perspectives.

## Supplementary information

**Additional file 1.** Semistructured interview guide

## Data Availability

The datasets generated and analysed during the current study are not publicly available due to risk of compromising individual confidentiality, but minimal dataset can be made available (in Norwegian) from the corresponding author on reasonable request, and with permission of DPOs at the respective hospitals.

## Abbreviations

CI: Confidence interval
OT: Operating theatre
SSC: Surgical safety checklist
WHO: World Health Organisation
