# Supplementary material for: How does the WHO Surgical Safety Checklist fit with existing perioperative risk management strategies? An ethnographic study across surgical specialties
Source: BMC Health Serv Res. 2020 Feb 12;20:111. doi: 10.1186/s12913-020-4965-5 (PMC7017532; doi:10.1186/s12913-020-4965-5)
Supplement: Supplementary file 1 — Additional file 1. Semistructured interview guide [file 12913_2020_4965_MOESM1_ESM.pdf]

INTERVIEW GUIDE

Interview number:___ **Date:________**

Setting:____

Interview participant (profession):____

Opening information to establish relationship with participants:
- Information on protection of anonymity of interview participants
- Clarification on role of the interviewer
- Clarification of the purpose of the study

Topic 1: World Health Organization`s Surgical Safety Checklist:
The SSC has been introduced as a safety tool to enhance perioperative teamwork and information exchange, by systematically reviewing critical patient factors before the induction of anaesthesia, before the incision of the skin, and before the patient leaves the operating facility.

As (the relevant profession):
- In your opinion, do you think the SSC work as intended at your surgical unit?
  - How?
  - Why?

- Can you describe a situation in which using the SSC has been useful or positive?
  - How?
  - Why?
  - Any experiences in relation to specific perioperative work processes?

- Can you describe a situation in which using the SSC has been difficult?
  - How?
  - Why?
  - Any experiences in relation to specific perioperative work processes?

Topic 2: Perioperative teamwork:
In the following, I will ask questions related to local team work- and communication.
As (the relevant profession):

- How do you experience that the SSC influence the perioperative teamwork?
    - How?
    - Why?

- Do you have any experiences of this in relation to "Sign In»?
    - (Issues and «patterns» of communication?)

- Do you have any experiences of this in relation to "Time Out»?
    - (Issues and «patterns» of communication?)

- Do you have any experiences of this in relation to "Sign Out»?
    - (Issues and «patterns» of communication?)

- Have you experienced that the SSC may influence your professional role in the perioperative teamwork?
    - How?
    - Why?

- How do you think that the SSC influence or contribute to patient safety at your surgical unit?
    - Why?

Closing questions:

- Is there anything you would like to add, that you believe is of importance in relation to the topics we have discussed?
    - The Surgical safety checklist and hospital compliance data?
    - Perioperative teamwork?
    - Specific perioperative work-processes?

- Do you have any thoughts or feedback on this interview?

Thank you for your participation!
